# Gastrotomy—(A Case of Ovarian Dropsy, Removal of Sac and Fatal Termination)

**Published:** 1850-11

**Authors:** A. H. Grimshaw

**Affiliations:** Wilmington, Del., Member of the American Medical Association


*Gastrotomy*—(*A case of Ovarian Dropsy, removal of sac and fatal termination.*) By A. H. Grimshaw, M. D., of Wilmington, Del., Member of the American Medical Association.

I consider that a man does not perform his duty to his fellow practitioners when he only publishes his successful cases, and, although I should have preferred to report this as a successful case, nevertheless, acting honestly and conscientiously, I give it as it occurred.

Edith Borkman, æt. 37 years, who has borne several children, one since the commencement of her dropsy, came into the Alms House of this county, Nov. 16th, 1848. She was then in bad health, cachectic, emaciated, and with great distension of the abdomen; there was also an immense ulcer occupying the umbilical region, and destroying all traces of umbilicus. Her case being diagnosticated ovarian dropsy, to relieve urgent symptoms she was tapped by the attending physician. The ulcer healed almost immediately after this tapping, in consequence of the removal of the distension; her health also improved. After this, she menstruated once, and left the house, remaining out till March 12th, 1849, since which time, till the day of her death, she continued in the house, and was tapped eight times; at each tapping, about four gallons and a half of fluid were drawn off, sometimes straw colored, at others, chocolate-like.

She came under my care in July, and I performed the last tapping about ten days previously to the operation about to be described, and drew off five gallons of fluid. The operations of tapping were only resorted to when the woman's comfort and health began to suffer from the distension. Between the first two tappings, eight months elapsed; after this, about four weeks was the average. Her health was good, appetite excellent, and spirits cheerful. Before coming under my care, I had mentioned to her that such tumours had been extracted, and as soon as I took charge, she requested me to perform an operation for her relief. Her case was one upon which I deliberated seriously, and one which was inspected by my medical friends of this city, both before and after the tappings. Before the last, she measured 52 inches around the hips, and 39 from the scrobiculus cordis to the pubis.

Upon looking into all the authorities within my reach, and, as the patient herself expressed it, knowing that she must inevitably die, and that within a comparatively short time, if an operation would not prolong her life, I deemed an operation for extraction to be justifiable. I shall not attempt to prolong this article by arguing this point. Dr. Atlee has demonstrated the feasibility and justifiability of the operation, in the "Amer. Med. Journal," but let me give one quotation from Chelius' Surgery. "If the operation is to become established, of which I have the strongest doubts, it must be confined to examples of the malady where tapping has been already so often performed as to preclude, from the experience of similar cases, any idea that it can ever be dispensed with, and when we are confident that great suffering must lead to early death." Again, "In such cases, if the diagnosis excludes the belief that there are serious adhesions, or malignant and solid growths complicating the tumour, and if the patient strongly desires it, the operation is defensible."

Late reports prove that it is defensible even when we have "serious adhesions," even when "solid growths" exist. In this case there was ovarian dropsy; we did not fear, but expected to meet serious adhesions; the patient "strongly desired it," even after I had told her that *one half* die under the operation.

On Wednesday, Sept. 4th, 1850, at 12 M., the pulse being 80 per minute, I commenced the operation. Present, Drs. Thomson, Askew, Bush, Porter, Baker, Barstow, and my colleague, Dr. Wilson, together with several medical students. The patient was under the influence of ether. I made an incision about 12 inches long, from a point about three inches to the right of the umbilicus, (to avoid the cicatrix) to the linea alba, above the pubis. This cut allowed a considerable quantity of water to escape. The parietes of the abdomen, I should mention, were œdematous, and the fluid had separated the fascia from the integuments to a considerable extent; there was also a thick layer of fat over the whole abdomen. In attempting to penetrate the fibrous layer, now exposed to view, my knife punctured the sac, which was adherent at this point and quite tense; this allowed the contents of the sac to escape. I then attempted to find the point of junction of sac and fascia, and imagining I had done this, separated the fascia on each side of the incision, before detecting the error. There was no appearance of muscular fibre. At last when I had almost despaired of succeeding in extracting the sac, on account of these difficulties, upon deepening my incision near the pubis, a thin layer of muscular fibres was severed, the remains of the pyramidalis. Penetrating at this point, I was enabled to detach the sac, with comparative ease, the adhesions all being round the point at which I had first entered, being an extension of the disease of the integuments, involving all the subjacent structures.

The tumour always had retained a position on the right side; after tapping, small tumours could be detected attached to the sac. It was in reality a dropsy of the left ovary, the sac, when unattached had fallen over to the right side, twisting the pedicle, or broad ligament. I applied three ligatures to this and cut it off. The Fallopian tube appeared to be healthy. The uterus to all appearance healthy, confirming our opinion formed by an examination per vaginam. The right ovary was larger than natural, more solid and whitish coloured. We closed up the wound; the patient was exceedingly feeble, and still under the influence of ether, and refused to take stimulants. She had lost a large quantity of blood, part from the wound in integuments, and part from several small vessels wounded in attempting to separate the fascia; torsion was used to each vessel as soon as it was wounded.

The wound was dressed and the patient put to bed. There was great restlessness and complaint of heat of body, also great thirst, pulse feeble and apparently rising; stimulants were administered freely, with an anodyne. Until 4 P. M., although the case presented a decidedly unfavorable appearance, we had hope. After 4 o'clock she sank fast, and died at 5 o'clock 10 minutes.

The next morning, upon examining the cavity of the abdomen, we found that about four ounces of blood had escaped into the cavity of the peritoneum, and was mixed with some of the fluid from the sac. The right ovary, upon cutting it open, was found to contain several small cysts.

Notwithstanding the sudden and unfortunate termination of this case, I should give my voice for the operation. I would beg the medical gentlemen who dread the knife, and shudder at the unfortunate termination of a surgical case, to take the scalpel and operate on their *dead patients;* let them, after dissection has revealed to them mistakes in diagnosis, hidden maladies not "dreamed of in their philosophy," be disposed to be charitable, and acknowledge that they have not only failed to relieve their patients but sometimes have not applied the correct treatment. Rather attribute the fatal issue of this operation to my want of skill, than to the unjustifiability of the operation. I consider it more proper than tying the primitive carotid for the cure of erectile tumors on the head and face, and I shall under like circumstances, if my patient will consent, operate again.

[We commend the honesty and moral courage of our correspondent in thus reporting his unsuccessful case, although we regret its termination. If all were equally fearless, this, as well as some other heroic operations, would lose much of their eclat, a great portion of which is to be traced to the fact that the *whole truth* is not divulged.—Ed. Exam.]

